# Assessing Sexual Health Knowledge, Attitude, and Perception Among Adolescents: A Pre-post Intervention Study in Varanasi, India

**DOI:** 10.7759/cureus.111773

**Published:** 2026-06-29

**Authors:** Vineet Kumar Pathak, Prayag Khandelwal, Ravi Shankar, Ravpreet Kaur

**Affiliations:** 1 Community Medicine, Banaras Hindu University Institute of Medical Sciences, Varanasi, IND

**Keywords:** adolescent and sexual health, attitude, education intervention, knowledge assessment, lgbt health

## Abstract

Introduction: Adolescents today are undergoing physical, emotional, and social changes to grow up as healthy and productive individuals. They have very specific needs that must be met, chief among which is sexual reproductive health information. However, even when information is supplied to adolescents, much of the information can be incorrect or even be used to encourage adolescents to practice sexual health adversely. It is thus the aim of this study to assess the level of knowledge, attitudes, and perceptions that adolescents have with regard to sex education and to assess the impact of a specific sex education program in the rural and urban settings of Varanasi, India.

Materials and methods: The study was conducted using a single-group, pre-post interventional approach. A total of 370 adolescents (11-18 years) studying in schools and colleges of rural and urban areas of Varanasi, Uttar Pradesh, were selected as the study population. The present study assessed adolescents' baseline sexual health knowledge and their attitudes and perceptions regarding sex education and examined the immediate changes in these outcomes following a structured sex education session. A single session of sex education was conducted for the study participants after 60 minutes of baseline questionnaire, and the same questionnaire was administered to them immediately after the intervention. Paired comparisons were made using appropriate statistical tests.

Results: The findings were further summarized to reveal that 70.0% of the adolescents studied had low knowledge of sex education, 41.4% had a low attitude towards sex education, and 61.1% had a low perception of sex education. However, all these scores indicated significant improvement in the knowledge, attitude, and perception (KAP) of the adolescents towards sex education following the intervention. The mean knowledge scores on sex education increased from 1.75±1.20 at the baseline to 4.53±0.85 at the end line. Similarly, the mean scores for attitude towards sex education increased from 3.59±1.60 at the baseline to 5.11±0.90 at the end line. The mean scores for perception on sex education increased from 1.38±1.01 at the baseline to 2.20±0.86 at the end line. The overall mean KAP scores increased from 6.72±2.61 at the baseline to 11.84±1.85 at the end line, with a mean gain of 5.11 points (95% CI: 4.79-5.44; p<0.001). The awareness of the adolescents regarding issues such as awareness of the lesbian, gay, bisexual, and transgender (LGBT) community increased from 9.2% at the baseline to 84.1% at the end line.

Conclusion: Thus, there was a significant change in KAP of adolescents with regard to sex education through the said intervention. The findings of the study would help in promoting and integrating into the school and college curriculum of the country, in general, and the state of Uttar Pradesh, in particular, sex education programs that are culturally appropriate to equip adolescents to make informed decisions regarding their sexual and reproductive health.

## Introduction

Adolescence is a critical developmental stage of human life during which physical growth, cognitive maturation, emotional development, and social learning occur simultaneously. During this period, adolescents gradually develop autonomy, establish peer relationships, explore identity, and begin to make independent health-related decisions. Health behaviours adopted during adolescence may influence present well-being and may also continue into adulthood, thereby affecting long-term health outcomes [1].

Sexual and reproductive health is an important component of adolescent health that requires focused attention. In many settings, adolescents face barriers in accessing accurate information and services related to puberty, menstruation, sexuality, contraception, sexually transmitted infections, consent, and personal safety. These barriers are often shaped by sociocultural norms, gender expectations, stigma, and discomfort within families and communities regarding discussion of sexuality-related issues [2].

Because of these restrictions, adolescents may seek information from alternative sources, such as peers, print and electronic media, the internet, and other digital platforms. Information obtained through these informal sources may be incomplete, inaccurate, or misleading. Such misinformation can reinforce myths and stigma related to sexuality and reproductive health, promote unhealthy gender attitudes, and delay appropriate care-seeking from adolescent-friendly health services [3].

The Comprehensive Sexuality Education (CSE) proposed by the United Nations Educational, Scientific and Cultural Organization (UNESCO) is a curriculum-based educational approach that equips children and young people with scientifically accurate, age-appropriate, and culturally relevant knowledge, attitudes, values, and life skills. It helps learners understand human development, relationships, sexuality, gender, rights, consent, safety, and sexual and reproductive health, while promoting dignity, respect, equality, responsibility, and informed decision-making [4].

CSE does not promote early sexual activity. The World Health Organization (WHO) states that high-quality sexuality education is associated with delayed sexual debut, reduced sexual risk-taking, and increased use of contraception, while it does not increase sexual activity, risky sexual behaviour, or rates of sexually transmitted infections [5]. Evidence from international reviews also supports the effectiveness of curriculum-based sexuality and human immunodeficiency virus (HIV) education programmes. A study has reported that several well-designed sex and HIV education programmes improved knowledge, attitudes, and protective behaviours among young people, and many programmes delayed sexual initiation or reduced sexual risk behaviour [6]. Similarly, a systematic review and meta-analysis from low- and middle-income countries found that school-based sex education and HIV prevention interventions improved HIV-related knowledge and self-efficacy and showed beneficial effects on condom use and other preventive behaviours among young people [7].

Adolescent health has emerged as an important public health priority in India. The Government of India launched the Rashtriya Kishor Swasthya Karyakram (RKSK) to address adolescent health through a comprehensive framework that includes sexual and reproductive health, nutrition, mental health, substance misuse, injuries and violence, non-communicable diseases, counselling, and adolescent-friendly health services [8].

Schools and colleges are important platforms for delivering health education and health promotion interventions to adolescents. However, many adolescent students remain hesitant to discuss sexual and reproductive health concerns with parents, teachers, guardians, and healthcare providers because of sociocultural barriers, lack of trained educators, discomfort among adults, and inadequate adolescent-friendly communication. Designing acceptable, feasible, sustainable, and effective school- or college-based interventions is therefore essential. Such interventions can be delivered through trained teachers, health workers, educators, and healthcare providers. Before planning these interventions, it is important to assess adolescents' knowledge, attitudes, and perceptions (KAP) regarding sexuality education and sexual and reproductive health [9].

Knowledge reflects the factual information adolescents possess regarding puberty, reproductive health, contraception, sexually transmitted infections, consent, and personal safety. Attitude reflects their positive or negative orientation towards these issues. Perception reflects their views regarding the relevance, acceptability, appropriateness, and usefulness of sexuality education. These dimensions are interrelated but distinct, and assessment of all three is important before developing adolescent health education programmes. Educational institutions are suitable settings for imparting sexuality education because they provide a structured, continuous, and accessible platform for reaching adolescents. Health education delivered through schools and colleges can help adolescents acquire correct knowledge, develop healthy attitudes, and build skills required for responsible decision-making regarding their health and well-being.

Locally generated evidence is necessary because adolescents' KAP are influenced by family environment, school culture, gender norms, urban or rural background, and exposure to media. Indian evidence indicates that adolescents often have inadequate knowledge of sexual and reproductive health, although many students show favourable attitudes towards the inclusion of sex education in the school curriculum. Adolescents' participation in health-related learning and decision-making is important because this age group is gradually developing the capacity to understand health information, communicate needs, and act upon guidance received from families, schools, communities, and health systems [10].

Therefore, a study conducted in Varanasi would be useful for understanding the local context and for developing age-appropriate, culturally acceptable, and scientifically sound school- or college-based health education programmes. Such evidence may help in improving adolescent knowledge, correcting misconceptions, promoting healthy attitudes, and strengthening acceptance of sexuality education among school- and college-going adolescents.

The main objective of the present study was to assess the KAP regarding sex education among school- and college-going adolescents in Varanasi. The study also aimed to examine the association of adolescents' KAP with selected sociodemographic and educational characteristics. The study also attempted to assess the impact of a structured health education programme on KAP regarding sex education among school- and college-going adolescents.

## Materials and methods

An institution-based, single-group, pre-post interventional study was conducted among adolescents enrolled in selected schools of Varanasi, Uttar Pradesh, India. The study included institutions from both rural and urban areas to ensure representation of adolescents from different residential and educational backgrounds. The study period was 12 months, and data were collected from April to July, 2025. The study was designed to assess baseline KAP regarding sex education and to evaluate the immediate change following a structured educational intervention.

The participants comprised adolescents enrolled in educational institutions within the selected rural and urban study areas of Varanasi. Institutions were considered eligible if they were located in a selected study area, enrolled students within the study age range, had sufficient potentially eligible students in the included grades, and provided written administrative permission. Classes that had recently received a formal sexual or reproductive health education programme were excluded from the study.

This study used multistage non-probability sampling for the selection of participants from educational settings, both rural and urban. The study used an accessible institutional frame of 65 educational institutions (33 from urban and 32 from rural settings). Educational institutions were considered eligible if they were located within the selected rural or urban study areas of Varanasi, enrolled students aged 11-18 years in the educational levels included in the study, and had sufficient numbers of potentially eligible students. Institutions exclusively serving students outside the prespecified age range, or those unable to accommodate the baseline assessment, educational session, and immediate post-intervention assessment, were excluded. 

From the accessible institutional frame, institutions were approached purposively based on feasibility, administrative permission, rural-urban location, and availability of eligible students in classes 6th-12th. Out of the total, 10 institutions were approached for study, and permission was granted by the administrative head of five institutions (three from urban and two from rural settings). Students were eligible for enrolment if they were aged 11-18 years, were enrolled in one of the included classes at a participating educational institution, were present during the baseline data collection visit, were able to understand and complete the questionnaire in the language in which it was administered, and completed the applicable written consent and assent procedures. Students were excluded if they reported having recently attended a formal sexual or reproductive health education session before the baseline assessment or were unable or unwilling to complete the questionnaire.

All students from 6th to 12th standard who were present in the institution on the researcher's first visit to the institution were invited to participate in the study. A total of 391 adolescents completed the baseline assessment by the researcher. Paired analysis was conducted with the 370 adolescents who had complete pre-intervention and post-intervention assessments.

The sample size was calculated using an expected prevalence of adequate awareness regarding sex education of 62%, based on previous literature [11], with 95% confidence level and 5% absolute precision. Using the formula 'n = Z²pq/d²', the minimum required sample size was approximately 361. This was rounded to 370 for field feasibility and to ensure adequate paired data for pre-post analysis.

Data were collected using a self-administered, semi-structured questionnaire adapted from a previously published adolescent sex-education study [11]. The questionnaire consisted of four sections: socio-demographic characteristics, knowledge regarding sex education, attitude towards sex education, and perception regarding sex education. Socio-demographic variables included age group, sex, education category, syllabus/board, and residence. The knowledge section assessed awareness of sex education concepts; sexually transmitted diseases; the lesbian, gay, bisexual, and transgender (LGBT) community; good touch and bad touch; and menstruation/menstrual hygiene. The attitude section assessed support for school-based sex education, preferred age and frequency of teaching, comfort in discussing sexual health topics, perceived readiness of the education system, and perceived role of sex education in reducing sexual harassment. The perception section assessed comfort with teachers as educators and the institutional arrangement of sex education classes. It was field-pretested among 10 adolescents, comprising five males and five females, from a population similar to the study population; these participants were not included in the main analysis. Pretesting assessed comprehension, acceptability, question sequence, and response options, and wording was modified where necessary. Responses with an unambiguous favourable or correct direction were coded as 1, while other responses were coded as 0. Domain and total scores were treated as exploratory summary indices rather than validated latent scales. Baseline Cronbach's alpha values were 0.368 for knowledge, 0.592 for attitude, 0.566 for perception, and 0.611 for the 14-item total KAP index. Because these values indicated limited internal consistency, item-level paired analyses were emphasized, and composite-score findings were interpreted cautiously.

After completion of the baseline questionnaire, participants attended a structured sex education session lasting approximately 45 minutes. The post-intervention questionnaire was administered immediately after completion of the session. Sessions were conducted in a classroom in groups of approximately 20 students. The session was delivered by a trained co-researcher using lectures (slides and posters as teaching and learning methods).

The session addressed pubertal and bodily changes, menstruation and menstrual hygiene, reproductive health, sexually transmitted infections, differentiation of good and bad touch, consent, personal safety, healthy relationships, and safe health practices. Content was developed using the CSE guideline [5] and adapted for age and cultural appropriateness. Printed handouts were provided regarding facts and myths.

To promote consistency across institutions, the facilitators used the same teaching-learning method and content, sequence of topics, key messages, and approximate time allocation for every session. Facilitators received training before data collection. Intervention delivery was monitored using a checklist. No additional educational session or individualized counselling was provided before the post-intervention assessment. The questionnaire was immediately readministered after the session.

The primary outcome was the change in total KAP score from pre-intervention to post-intervention. Secondary outcomes included changes in KAP domain scores and item-wise changes in favourable responses.

Knowledge was scored using five binary indicators: comprehensive concept of sex education, awareness of sexually transmitted diseases, awareness of the LGBT community, ability to differentiate good touch and bad touch, and awareness of menstruation and menstrual hygiene. Attitude was scored using six favourable indicators: support for teaching sex education in schools, support for discussion by 16-17 years or earlier, preference for repeated teaching, comfort discussing with parents or guardians, perceived readiness of the education system, and belief that sex education can reduce sexual harassment. Perception was scored using three indicators: comfort if taught by teachers, institutional arrangement of classes, and receipt of repeated classes. The total KAP score ranged from 0 to 14. Scores were categorized as low, moderate, and high using percentage cut-offs of <50%, 50-75%, and >75% of the maximum possible score.

Data were entered, cleaned, and analysed using Stata (version 12; StataCorp LP, College Station, TX) [12]. Categorical variables were summarized as frequencies and percentages. Continuous scores were summarized as mean, standard deviation, and 95% confidence interval. Baseline socio-demographic characteristics were presented for the paired analytic sample. 

The study was approved by the Institutional Ethics Committee (IMS/IEC/2025/7829). Before enrolment, written informed consent was obtained from a parent or legal guardian of each participant aged 11-17 years, together with written age-appropriate assent from the adolescent. Participants aged 18 years provided written informed consent on their own behalf. Questionnaires were coded without personally identifying information, and all study records were handled confidentially.

Item-level outcomes were dichotomized as favourable/correct versus other responses. Pre-post changes in these paired binary outcomes were assessed using McNemar's exact test, calculated from the discordant pairs using the exact binomial distribution. For the multivariable linear regression model, the linear functional form was assessed by testing an additional quadratic baseline KAP term. Residual normality was evaluated using the Jarque-Bera test, homoscedasticity using the Breusch-Pagan test, and multicollinearity using variance inflation factors. Standardized residuals, leverage, and Cook's distance were examined for influential observations. Because residual non-normality and mild heteroskedasticity were detected, coefficient inference was based on HC3 heteroskedasticity-consistent robust standard errors and 95% confidence intervals. A sensitivity analysis excluding observations with Cook's distance greater than 4/n was also performed.

A multivariable linear regression model was used to identify predictors of total KAP score gain. Total KAP score gain was entered as the dependent variable, while baseline total KAP score, age group, sex, education category, syllabus/board category, and residence were entered as independent variables. Adjusted beta coefficients, standard errors, 95% confidence intervals, p values, and model R² were reported. A p-value <0.05 was considered statistically significant.

## Results

A total of 370 adolescents with complete paired pre-intervention and post-intervention responses were included in the final analysis. The baseline socio-demographic characteristics of the participants are presented in Table 1. Nearly 187 (50.5%) participants belonged to the 13-14 years age group, followed by 140 (37.8%) in the 11-12 years age group and 43 (11.6%) in the 15-18 years age group. Males constituted 213 (57.6%) of the study population, while females accounted for 157 (42.4%). Regarding educational status, 219 (59.2%) participants were studying in middle school, whereas 151 (40.8%) were enrolled in secondary school. A majority of the participants, 220 (59.5%), were studying under the central board syllabus, while 150 (40.5%) belonged to the state board. Both urban and rural adolescents were represented in the study, with 203 (54.9%) participants residing in urban areas and 167 (45.1%) residing in rural areas. Overall, the sample included adolescents from diverse age groups, educational backgrounds, academic boards, and residential settings.

**Table 1:** Socio-demographic characteristics of the study participants (paired analytic sample, N=370) Middle school included classes 6-8, and secondary school included classes 9-12. Table Credit: Vineet Kumar Pathak

Variable	Category	n (%)
Age	11-12 years	140 (37.8)
	13-14 years	187 (50.5)
	15-18 years	43 (11.6)
Sex	Male	213 (57.6)
	Female	157 (42.4)
Education	Middle School	219 (59.2)
	Secondary School	151 (40.8)
Syllabus/board	State	150 (40.5)
	Central	220 (59.5)
Residence	Urban	203 (54.9)
	Rural	167 (45.1)

The baseline knowledge score was low, with a mean score of 1.75±1.20 out of 5 (Table 2). Overall, 259 participants (70.0%) were classified as having low knowledge, whereas only 23 participants (6.2%) had high knowledge. The mean attitude score was 3.59 ± 1.60 out of 6; however, 153 participants (41.4%) were still categorized as having low attitude scores. Baseline perception was also limited, with a mean score of 1.38±1.01 out of 3, and 226 participants (61.1%) fell in the low-perception category. The mean total KAP score at baseline was 6.72±2.61 out of 14, with only 22 participants (5.9%) achieving a high overall KAP level.

**Table 2:** Baseline knowledge, attitude, and perception status of participants Knowledge max=5, attitude max=6, perception max=3, total knowledge, attitude, and perception (KAP) max=14. Level cut-offs: low (<50%), moderate (50-75%), and high (>75% of maximum score)

Domain	Mean score ± SD	95% CI for mean	Low n (%)	Moderate n (%)	High n (%)
Knowledge	1.75 ± 1.20	1.63 to 1.87	259 (70.0)	88 (23.8)	23 (6.2)
Attitude	3.59 ± 1.60	3.43 to 3.76	153 (41.4)	94 (25.4)	123 (33.2)
Perception	1.38 ± 1.01	1.28 to 1.48	226 (61.1)	73 (19.7)	71 (19.2)
Total KAP	6.72 ± 2.61	6.46 to 6.99	219 (59.2)	129 (34.9)	22 (5.9)

**Figure 1:**
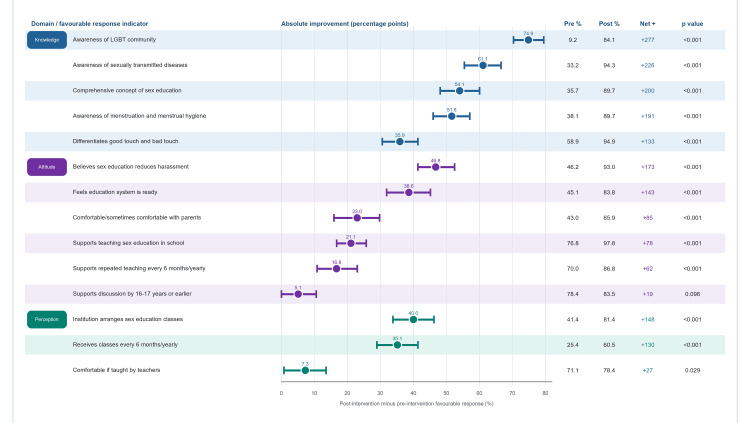
Item-wise pre-post improvement forest plot of favourable responses

Item-wise pre-post changes in favourable responses show marked improvements across most knowledge indicators after the educational intervention (Table 3, Figure 1). The greatest absolute increase was seen in awareness of the LGBT community, which improved from 34 participants (9.2%) before intervention to 311 participants (84.1%) after intervention, corresponding to a gain of 74.9 percentage points. Awareness of sexually transmitted diseases increased from 123 (33.2%) to 349 (94.3%), while comprehensive understanding of sex education increased from 132 (35.7%) to 332 (89.7%). Substantial improvements were also observed for awareness of menstruation and menstrual hygiene, ability to differentiate good touch and bad touch, support for repeated sex education, comfort discussing such topics with parents or guardians, and institutional exposure to sex education. Most item-wise changes were statistically significant on McNemar testing (p<0.001). The only item that did not show a statistically significant change was support for discussion of sexual and reproductive health by 16-17 years or earlier, which increased from 78.4% to 83.5% (p=0.096).

**Table 3:** Item-wise pre-post changes in favourable responses after an educational intervention LGBT: lesbian, gay, bisexual, and transgender

Domain	Indicator	Pre n (%)	Post n (%)	Change, percentage points	Improved n	Worsened n	McNemar p
Knowledge	A correct, comprehensive concept of sex education	132 (35.7)	332 (89.7)	54.1	220	20	<0.001
Knowledge	Awareness of sexually transmitted diseases	123 (33.2)	349 (94.3)	61.1	232	6	<0.001
Knowledge	Awareness of the LGBT community	34 (9.2)	311 (84.1)	74.9	279	2	<0.001
Knowledge	Differentiates good touch and bad touch	218 (58.9)	351 (94.9)	35.9	143	10	<0.001
Knowledge	Awareness of menstruation and menstrual hygiene	141 (38.1)	332 (89.7)	51.6	201	10	<0.001
Attitude	Supports teaching sex education in school	284 (76.8)	362 (97.8)	21.1	85	7	<0.001
Attitude	Supports discussion by 16-17 years or earlier	290 (78.4)	309 (83.5)	5.1	68	49	0.096
Attitude	Supports repeated teaching every 6 months/yearly	259 (70.0)	321 (86.8)	16.8	101	39	<0.001
Attitude	Comfortable/sometimes comfortable discussing with parents	159 (43.0)	244 (65.9)	23.0	136	51	<0.001
Attitude	Feels the education system is ready	167 (45.1)	310 (83.8)	38.6	175	32	<0.001
Attitude	Believes sex education can reduce sexual harassment	171 (46.2)	344 (93.0)	46.8	181	8	<0.001
Perception	Comfortable if taught by teachers	263 (71.1)	290 (78.4)	7.3	85	58	0.029
Perception	The institution arranges sex education classes	153 (41.4)	301 (81.4)	40.0	171	23	<0.001
Perception	Receives classes every 6 months/yearly	94 (25.4)	224 (60.5)	35.1	160	30	<0.001

The mean knowledge score increased from 1.75±1.20 to 4.53±0.85, with a mean paired gain of 2.78 points (95% CI: 2.63-2.92; p<0.001). This represented a large intervention effect (Cohen's dz=1.93). The mean attitude score increased from 3.59±1.60 to 5.11±0.90, with a mean gain of 1.51 points (95% CI: 1.32-1.70; p<0.001). The mean perception score increased from 1.38±1.01 to 2.20±0.86, with a mean gain of 0.82 points (95% CI: 0.69-0.96; p<0.001). The total KAP score increased from 6.72±2.61 at baseline to 11.84±1.85 after intervention, with a mean paired increase of 5.11 points (95% CI: 4.79-5.44; p<0.001) and a large effect size (Cohen's dz=1.61).

Table 4 presents the association of baseline knowledge, attitude, and perception levels with selected socio-demographic variables. Table 5 shows the baseline distribution of KAP levels across selected socio-demographic characteristics. For knowledge, sex was the only statistically significant factor (p<0.001), with low knowledge more frequent among females than males; age and residence showed borderline but non-significant associations. For attitude, significant associations were observed with age (p=0.003) and sex (p<0.001), indicating a more favourable baseline attitude among older adolescents and males, while education, syllabus/board, and residence were not statistically significant. For perception, significant associations were observed with sex (p=0.005), syllabus/board (p<0.001), and residence (p<0.001), suggesting that baseline perception varied more strongly by educational board and place of residence than baseline knowledge did. Overall, the expanded table demonstrates that socio-demographic variation was not limited to knowledge alone; attitude and perception also differed across key subgroups, supporting the need for subgroup-sensitive planning of adolescent sex education interventions.

**Table 4:** Association of baseline knowledge, attitude, and perception levels with selected socio-demographic variables

Domain	Variable	Category	Total n	Low n (%)	Moderate n (%)	High n (%)	Chi-square p
Knowledge	Age	11-12 years	140	105 (75.0)	25 (17.9)	10 (7.1)	0.077
		13-14 years	187	126 (67.4)	48 (25.7)	13 (7.0)	
		15-18 years	43	28 (65.1)	15 (34.9)	0 (0.0)	
Knowledge	Sex	Male	213	134 (62.9)	66 (31.0)	13 (6.1)	<0.001
		Female	157	125 (79.6)	22 (14.0)	10 (6.4)	
Knowledge	Education	Middle School	219	153 (69.9)	50 (22.8)	16 (7.3)	0.540
		Secondary School	151	106 (70.2)	38 (25.2)	7 (4.6)	
Knowledge	Syllabus/Board	State	150	101 (67.3)	42 (28.0)	7 (4.7)	0.211
		Central	220	158 (71.8)	46 (20.9)	16 (7.3)	
Knowledge	Residence	Urban	203	150 (73.9)	45 (22.2)	8 (3.9)	0.074
		Rural	167	109 (65.3)	43 (25.7)	15 (9.0)	
Attitude	Age	11-12 years	140	67 (47.9)	29 (20.7)	44 (31.4)	0.003
		13-14 years	187	77 (41.2)	55 (29.4)	55 (29.4)	
		15-18 years	43	9 (20.9)	10 (23.3)	24 (55.8)	
Attitude	Sex	Male	213	62 (29.1)	49 (23.0)	102 (47.9)	<0.001
		Female	157	91 (58.0)	45 (28.7)	21 (13.4)	
Attitude	Education	Middle School	219	99 (45.2)	48 (21.9)	72 (32.9)	0.104
		Secondary School	151	54 (35.8)	46 (30.5)	51 (33.8)	
Attitude	Syllabus/Board	State	150	52 (34.7)	39 (26.0)	59 (39.3)	0.062
		Central	220	101 (45.9)	55 (25.0)	64 (29.1)	
Attitude	Residence	Urban	203	75 (36.9)	60 (29.6)	68 (33.5)	0.075
		Rural	167	78 (46.7)	34 (20.4)	55 (32.9)	
Perception	Age	11-12 years	140	93 (66.4)	21 (15.0)	26 (18.6)	0.225
		13-14 years	187	110 (58.8)	39 (20.9)	38 (20.3)	
		15-18 years	43	23 (53.5)	13 (30.2)	7 (16.3)	
Perception	Sex	Male	213	121 (56.8)	39 (18.3)	53 (24.9)	0.005
		Female	157	105 (66.9)	34 (21.7)	18 (11.5)	
Perception	Education	Middle School	219	143 (65.3)	40 (18.3)	36 (16.4)	0.119
		Secondary School	151	83 (55.0)	33 (21.9)	35 (23.2)	
Perception	Syllabus/Board	State	150	62 (41.3)	37 (24.7)	51 (34.0)	<0.001
		Central	220	164 (74.5)	36 (16.4)	20 (9.1)	
Perception	Residence	Urban	203	96 (47.3)	50 (24.6)	57 (28.1)	<0.001
		Rural	167	130 (77.8)	23 (13.8)	14 (8.4)	

**Table 5:** Domain-wise pre-post score change after an educational intervention KAP: knowledge, attitude, and perception

Domain	Pre mean ± SD	Post mean ± SD	Mean change	95% CI of change	Cohen dz	p value
Knowledge	1.75 ± 1.20	4.53 ± 0.85	2.78	2.63 to 2.92	1.93	<0.001
Attitude	3.59 ± 1.60	5.11 ± 0.90	1.51	1.32 to 1.70	0.81	<0.001
Perception	1.38 ± 1.01	2.20 ± 0.86	0.82	0.69 to 0.96	0.64	<0.001
Total KAP	6.72 ± 2.61	11.84 ± 1.85	5.11	4.79 to 5.44	1.61	<0.001

In the multivariable linear regression analysis (Table 6), the model explained 67.2% of the observed variation in total KAP score change (R²=0.672). Diagnostic assessment found no evidence of departure from linearity (p=0.376) and no severe multicollinearity (maximum variance inflation factor=4.42). However, residual non-normality (Jarque-Bera p<0.001) and mild heteroskedasticity (Breusch-Pagan p=0.047) were detected; therefore, HC3 heteroskedasticity-consistent robust standard errors were used. After adjustment for age, sex, educational level, syllabus/board, and residence, a higher baseline total KAP score was associated with a smaller observed score gain (adjusted β=-0.939, robust SE=0.044, 95% CI: -1.024 to -0.853; p<0.001). None of the socio-demographic variables was independently associated with KAP score change. Thirteen observations exceeded Cook's distance screening threshold of 4/n; however, excluding these observations produced a nearly identical baseline-score coefficient (β=-0.941), indicating that the principal finding was not driven by influential observations.

**Table 6:** Multivariable linear regression predicting the total KAP score gain after the intervention Model summary: R² = 0.672; adjusted R² = 0.666; N = 370. Coefficient inference was based on HC3 heteroskedasticity-consistent robust standard errors. The dependent variable was the total KAP score change, calculated as the post-intervention score minus the baseline score.

Predictor	Adjusted β	HC3 robust SE	95% CI	p value
Baseline total KAP score	-0.939	0.044	-1.024 to -0.853	<0.001
Age 13-14 vs 11-12 years	-0.307	0.263	-0.823 to 0.208	0.243
Age 15-18 vs 11-12 years	-0.22	0.431	-1.065 to 0.625	0.610
Female vs male	0.134	0.284	-0.422 to 0.690	0.637
Secondary vs middle school	0.427	0.346	-0.250 to 1.105	0.217
Central vs state board	0.658	0.388	-0.103 to 1.419	0.090
Rural vs urban	0.115	0.341	-0.554 to 0.784	0.737

## Discussion

Participants demonstrated significantly higher KAP and total KAP scores at the immediate post-intervention assessment than at baseline. These findings indicate that the educational session was followed by immediate improvement in questionnaire responses. However, because this was a single-group pre-post study without a concurrent comparison group, the observed changes cannot be attributed solely to the intervention. Repeated exposure to the questionnaire, short-term recall, participant expectations, social-desirability effects, and other unmeasured factors may have contributed to the post-intervention responses.

Baseline sexual-health knowledge was limited in this study. Seven in every 10 participants were classified as having low knowledge (259/370, 70.0%), 88 (23.8%) had moderate knowledge, and only 23 (6.2%) had high knowledge. The mean baseline score was 1.75±1.20 out of 5, further indicating substantial gaps across the knowledge items assessed. These findings suggest that most participating adolescents entered the study with a limited understanding of the sexual-health topics included in the questionnaire. However, because participants were selected from consenting institutions using a non-probability approach, these proportions describe the study sample and should not be interpreted as population prevalence estimates for all adolescents in Varanasi. Though many of the adolescents who are willing to receive information on these aspects of human sexuality are not able to receive it due to a lack of formal programs of human sexuality education within schools, they rely on information and programs received through mass media, their peers, and books on the subject [13]. The reasons for this lack of knowledge are primarily due to the lack of formal programs of human sexuality education currently being provided within the formal education system in India. Moreover, there is resistance to discussing aspects of human sexuality within families of adolescents. Therefore, they are not able to receive information on these issues from their families. The same resistance to discussing issues of human sexuality is also observed within the larger society; therefore, there is also reluctance on the part of schools, as well as families and mass media, to discuss these issues with adolescents.

The most pronounced item-level changes concerned LGBT-related awareness and STIs. Favourable recognition of the LGBT-related item increased from 34/370 (9.2%) at baseline to 311/370 (84.1%) immediately after the intervention, representing an absolute increase of 74.9 percentage points. Recognition of the STI-related item increased from 123/370 (33.2%) to 349/370 (94.3%), an increase of 61.1 percentage points. These low baseline proportions identify substantial topic-specific knowledge gaps within the study sample. Such gaps may reflect limited previous access to structured, age-appropriate sexuality education, cultural discomfort surrounding discussions of sexual and gender diversity and infection risk, and reliance on peers or media for information [14,15]. The immediate changes are consistent with evidence that structured school-based sexual and reproductive health education can improve factual knowledge [16-19]. Nevertheless, participants with low baseline awareness had greater numerical scope for improvement, and the assessment occurred shortly after the session. Furthermore, each outcome was derived from a single dichotomized item. The LGBT-related finding therefore indicates immediate recognition of the concept assessed rather than comprehensive understanding, acceptance of sexual and gender diversity, or reduced stigma. Similarly, the STI item does not demonstrate detailed knowledge of transmission, prevention, symptoms, or care-seeking. Delayed assessment using validated multi-item measures would be required to determine the depth and retention of learning.

A positive change was also observed in the perception of the adolescents towards sex education. There was a significant increase in the favorable perception towards sex education. The adolescents were more inclined to accept sex education from teachers and schools and also demanded more of such programs. They were also in favor of conducting repeated programs for sex education. The main objective of sex education is to enable the adolescent to acquire communication skills, to take responsible and wise decisions with regard to his/her sexual behavior, to treat other individuals with dignity and respect, and to acquire knowledge and understanding of gender issues [17]. Thus, the present study also made a positive change in the attitude of the adolescents towards sex education and helped them to acquire knowledge regarding the same.

The scores for perception also improved, though not to the same extent as for knowledge. The adolescents felt comfortable enough to discuss sex education with teachers and would like to have such sessions set up within the institutions they attend. The role of schools as a potent health-promoting locus has been elucidated through this study conducted on adolescents in an Indian setup. As stated earlier in studies, adolescents in the study are hesitant to discuss sex related topics with their family members, but can discuss such topics with teachers of schools they attend. Thus, the study has underlined the importance of designing and implementing sex education programs in a culturally sensitive and evidence-based manner and delivering them on a regular basis through schools and teachers.

Baseline knowledge category differed significantly by sex. Among female participants, 125/157 (79.6%) were classified as having low knowledge compared with 134/213 male participants (62.9%; p<0.001). This finding indicates an association between sex and the distribution of baseline knowledge categories within the study sample; it should not be interpreted as a causal effect of sex or as direct evidence of a difference in mean knowledge scores. Previous literature from India and other low- and middle-income settings suggests that adolescent girls may encounter barriers to obtaining comprehensive sexual-health information, including cultural discomfort surrounding sexuality, restrictions on open discussion, concerns about respectability, and limited confidential access to appropriate information and services [11,18]. Differences in exposure to peers, digital media, school-based teaching, and parent or teacher communication may also contribute. However, the present study did not measure information sources, household communication, gender norms, internet access, or previous educational exposure. These explanations therefore remain hypotheses, and unmeasured confounding cannot be excluded. Future studies should directly assess these pathways and determine whether sex-related differences persist after adjustment for household, social, and educational factors.

The total KAP score at the baseline was the only significant predictor of improvement in KAP scores. The negative adjusted beta coefficient for this variable indicated that the adolescents with the worst KAP scores at the beginning of the study showed the greatest improvement in KAP scores following the intervention. This finding is important from a public health perspective as it indicates that the adolescents least informed about sexual health are most vulnerable to misinformation and therefore will benefit the most from receiving a program of sexuality education [19].

The baseline item-level findings identified knowledge gaps in several topics included in the questionnaire, particularly LGBT-related concepts, STIs, menstrual hygiene, the broader meaning of sex education, and differentiation between appropriate and inappropriate touch. More favourable responses were observed for these items immediately after the educational session. These findings suggest that structured, age-appropriate education may be a feasible approach for addressing specific informational gaps among students in similar settings. However, the study did not measure the prevalence of gender discrimination or sexual abuse, nor did it evaluate behavioural, service-use, or health outcomes. The results should therefore not be extrapolated to broader social conditions or interpreted as evidence that a particular policy would be effective at the population level. Controlled studies using representative sampling, validated measures, and longer follow-up are required to determine whether school-based programmes produce sustained improvements and can be integrated effectively within existing adolescent-health services.

This study has several strengths. The paired pre-post design enabled direct within-participant comparison of baseline and post-intervention responses, thereby reducing the influence of between-participant differences when estimating mean change. Complete paired observations were available for 370 adolescents from rural and urban settings. In addition to examining KAP and total KAP scores, individual questionnaire items were analysed to identify topic-specific gaps and immediate response changes. Paired statistical methods were used for binary and continuous outcomes, while mean differences, 95% confidence intervals, and standardized effect sizes described the magnitude and precision of the observed changes. Nevertheless, these strengths improve the assessment of within-participant change but do not compensate for the absence of a concurrent comparison group or establish that the educational session caused the observed differences.

The study had a few limitations. One of them was that there was no control group; thus, it could not be known whether any other external/unrelated factor(s) could have caused the changes observed in the participants. Because the post-intervention assessment was conducted immediately after the session, the observed changes may partly reflect short-term recall, repeated-testing effects, or participants' awareness of the study purpose. The study did not evaluate retention of knowledge, persistence of reported attitudes or perceptions, behavioural change, or health outcomes. Additionally, the regression models present a very high R^2 ^value, which may not reflect the true predictive model because of the nature of KAP as a study design. The study used a self-report instrument for data collection; thus, it could not rule out the possibility of social desirability bias in the study's findings. However, the findings of the study were consistent; thus, they supported the findings of the study.

The study found that adolescents in Varanasi lack critical knowledge, adequate positive attitudes, and appropriate perceptions regarding sexuality and health, and thus require school- and college-based health initiatives, which can provide adequate, culturally appropriate, evidence-based programs of sex education continuously.

## Conclusions

In this sample of school-going adolescents from selected rural and urban areas of Varanasi, baseline KAP regarding sex education and sexual health was limited. The structured educational session was followed by significant immediate improvements in sexual-health knowledge and in reported attitudes and perceptions regarding sex education. The largest item-wise gains were observed in areas where baseline awareness was the lowest, particularly awareness related to sexually transmitted infections and LGBT-related concepts, suggesting important topic-specific gaps in the study sample.

Adolescents also showed improved acceptance of schools and teachers as sources of sex education after the session. Participants with lower baseline KAP scores demonstrated greater observed improvement, indicating that structured educational sessions may be especially useful for adolescents with poorer baseline awareness. However, because this was a single-group pre-post study without a concurrent comparison group and the post-intervention assessment was conducted immediately after the session, the observed changes should be interpreted as short-term improvements in questionnaire responses rather than evidence of sustained behavioural change. Further controlled studies with representative sampling, validated outcome measures, and delayed follow-up are required to determine whether such improvements are retained over time and whether they translate into sustained attitudes, safer behaviours, or improved health outcomes. The findings support the need for culturally appropriate, age-sensitive, and evidence-based school-based sexuality education as part of adolescent health promotion.
